# Chronic kidney disease and underdiagnosis of renal insufficiency among diabetic patients attending a hospital in Southern Ethiopia

**DOI:** 10.1186/1471-2369-15-198

**Published:** 2014-12-15

**Authors:** Temesgen Fiseha, Mehidi Kassim, Tilahun Yemane

**Affiliations:** Department of Clinical Laboratory Science, College of Medicine and Health Science, Wollo University, Dessie, Ethiopia; Department of Biomedical Science, College of Public Health & Medical Sciences, Jimma University, Jimma, Ethiopia; Department of Hematology, College of Public Health & Medical Sciences, Jimma University, Jimma, Ethiopia

**Keywords:** Chronic kidney disease, Diabetes, Estimated glomerular filtration rate, Serum creatinine, Renal insufficiency

## Abstract

**Background:**

Diabetic patients with chronic kidney disease (CKD), as defined by a reduced glomerular filtration rate (GFR), are at greater risk for cardiovascular and renal events and mortality. The aim of this study was to determine the prevalence of CKD among diabetic patients attending a hospital in southern Ethiopia, and to assess underdiagnosis of renal insufficiency among those with normal serum creatinine.

**Methods:**

A total of 214 randomly selected diabetics attending the follow-up clinic at Butajira hospital of southern Ethiopia participated in this study during the period from September 1 to October 31, 2013. All patients completed an interviewer-administered questionnaire and underwent clinical assessment. The simplified Modification of Diet in Renal Disease (MDRD) and Cockroft-Gault (C-G) equations were used to estimate GFR (eGFR) from serum creatinine.

**Results:**

CKD, defined as eGFR < 60 ml/min/1.73 m^2^, was present in 18.2% and 23.8% of the study participants according to the MDRD and Cockcroft-Gault (C-G) equations, respectively. Only 9.8% of the total participants, and 48.7% (for the MDRD) and 37.3% (for C-G) of those with eGFR <60 ml/min/1.73 m^2^ had abnormal serum creatinine values, i.e. > 1.5 mg/dl. Normal serum creatinine was observed in 90.2% of participants attending the hospital. A large proportion of participants ranging from 38.9-56.5% have shown to have mild to moderate renal insufficiency (stage 2–3 CKD) despite normal creatinine levels. CKD, eGFR < 60 ml/min/1.73 m^2^, was found in 10.4 and 16.9% of participants with normal serum creatinine using the MDRD and C-G equations, respectively.

**Conclusion:**

CKD is present in no less than 18% of diabetics attending the hospital, but it is usually undiagnosed. A significant number of diabetics have renal insufficiency corresponding to stages 2–3 CKD despite normal creatinine levels. Therefore, GFR should be considered as an estimate of renal insufficiency, regardless of serum creatinine levels being in normal range.

## Background

Chronic kidney disease (CKD), characterized by a glomerular filtration rate (GFR) of less than 60 ml/min/1.73 m^2^, is a common and serious complication of diabetes [1]. Health consequences are substantial, with sufferers at increased risk of all-cause and cardiovascular mortality, progression to kidney failure, cardiovascular disease (CVD) and hospitalizations [2, 3]. For patients with diabetes and CKD, the risk of hypertension, anemia, malnutrition, bone and mineral disorders, and retinopathy is higher as compared to patients with normal renal function [4, 5]. There is an even higher prevalence of hypoglycemia due to decreased clearance of hypoglycemic agents or impaired renal gluconeogenesis [6].

Assessment of renal function is therefore important, as detecting CKD during its initial stages provides the opportunity for early therapeutic interventions to prevent or delay the onset of disease or complications and to improve outcomes. The GFR, an important component in the diagnosis of CKD, is accepted as the best index of overall kidney function [1, 7]. Measurement of GFR using exogenous filtration markers such as inulin or iothalamate are the definitive measures, but none of these are practical or economical for routine use [8].

Serum creatinine (SCr) has been proposed as an endogenous marker of GFR and used most frequently to assess renal function in clinical practices. However, SCr level, which is affected by factors other than the GFR, is insufficiently sensitive to detect CKD on its own, and might remain in the normal range despite the renal function is significantly impaired [9, 10].

Currently, an estimated GFR (eGFR) derived from prediction equations has been suggested as a simple, rapid and reliable means of assessing renal function, and have been shown to be more accurate in estimating GFR at levels below 60 ml/min/1.73 m^2^ [8, 11, 12]. Consequently, available guidelines recommend the annual measurement of SCr for eGFR in all adults with diabetes and the use of prediction equations that incorporate patient’s age, sex, race and body size for CKD diagnosis and staging renal insufficiency [1, 13–15].

Despite the fact that diabetic patients are more likely to have CKD, and thus suffer extra morbidity and mortality than those without the disease [16, 17]; there are virtually no published studies on the prevalence of CKD among diabetic patients in Ethiopia. More than this, many physicians relay on SCr as a measure of renal function rather than using for eGFR and interpret normal SCr levels as evidence of normal renal function. Therefore, the aim of this study was to estimate the prevalence of CKD among diabetic patients attending Butajira Hospital of southern Ethiopia, and to assess underdiagnosis of renal insufficiency among those with normal SCr.

## Methods

From 568 registered diabetic patients, 229 random samples of people aged 18 years and above were drawn out using a table of random numbers constructed from the register. A total of 214 diabetics attending the follow-up clinic of Butajira Hospital, southern Ethiopia from September 1 to October 31 2013 were included in this cross sectional study. Butajira Hospital is located in Butajira town, 130 km mid-south of the capital city of Ethiopia, Addis Ababa. The hospital registers and treats all diagnosed diabetic patients and provides primary diabetes patient care.

The study was approved by the Research Ethics Committee of Jimma University. An informed verbal consent was taken from all the patients enrolled. Participants, who were recruited after being screened and counseled by their clinicians, were adults (≥18 years) attending the diabetic clinic for follow-up. Patients were excluded if they were pregnant, hospitalized, have acute illnesses (fever), treated with dialysis and if they were not fasting.

During their visit participants were interviewed for collecting demographic and risk factor variables. Weight, height and blood pressure were measured at the time of the clinical examination performed. Body mass index (BMI) was calculated from weight (kg) in light clothing without shoes, and height (meters) without shoes. Obesity was defined as a BMI ≥30 kg/m^2^. Blood pressure (BP) was measured using a mercury sphygmomanometer in the right upper arm in the sitting posture, after a five minute rest and three measurements were averaged to be recorded. Hypertension was defined as systolic BP ≥140 mmHg or diastolic BP ≥90 mmHg or use of antihypertensive medication irrespective of the BP.

A blood sample was collected in the early morning after an overnight fasting. Biochemical analysis were done on HumaStar 80 clinical chemistry analyzer (Human Diagnostics, Germany) using kits supplied by HUMAN (Human Diagnostics, Germany). Fasting serum glucose was measured by enzymatic GOD-PAP method. SCr was measured using Jaffe kinetic method as mg/dl with calibration traceable to IDMS reference material NIST SRM 909B level 2.

### Measurement of kidney function

Kidney function was assessed according to the simplified version of the Modification of Diet in Renal Disease (MDRD) study equation [14]: 186 × SCr(mg/dl)^-1.154^ × age(years)^-0.203^ × 0.742 (if female) × 1.210 (as our population are Africans) and the Cockcroft-Gault (C-G) equation[15] normalized for the body surface area (BSA): (140-age) × Weight (Kg) × 0.86 (if female) × 1.73/72 × SCr (mg/dl) × BSA (m^2^).

All participants with eGFR < 60 ml/min/1.73 m^2^ at the first visit were advised to have their serum checked for creatinine two weeks after the first check-up. Staging of kidney function was based on the National Kidney Foundation- Disease Outcomes Quality Initiative (K/DOQI) classification. Stage 1 CKD was defined as normal or increased eGFR (eGFR ≥ 90 ml/min/1.73 m^2^). Mild, moderate, and severe renal insufficiency (RI) corresponding to stage 2, 3 and 4 CKD were defined as eGFR 60–89.9, 30–59.9, and, 15–29.9 ml/min/1.73 m^2^, respectively. For the purposes of this study, CKD was defined as eGFR <60 ml/min/1.73 m^2^ (stages 3–5 CKD)[1, 18].

### Statistical analysis

The data was entered in to “EpiInfo version 3.1” and was analyzed using SPSS version 20.0 statistical software. Data were expressed as means ± standard deviation (SD) or percentage. Chi-square (x^2^) test was used to compare proportions. Multivariate logistic regression was used to calculate adjusted odds ratios (OR) and the corresponding 95% confidence intervals (CI). P value < 0.05 was used to indicate statistical significance.

## Results

### Demographic and clinical characteristics of participants

A total of 214 diabetic patients participated in the study, 123(57.5%) males, and 114 (53.3%) type 2 diabetics. Mean age of participants was 45 ± 14.5 years, ranging from 19 to 90 years and 40 (18.7%) of them were above 60 years old. Mean body mass index (BMI) was 25.26 ± 4.35Kg/m^2^, and 31 (14.5%) of them were obese (BMI ≥30 kg/m^2^). The mean systolic and diastolic BP of participants was 121 ± 17 and 79 ± 10 mmHg, respectively and 113 (52.8%) were hypertensive. Mean fasting serum glucose (FSG) was 172.85 ± 84.94 mg/dl. Mean serum creatinine (SCr) was 1.07 ± 0.33 mg/dl. The mean eGFR values according to the MDRD and C-G equations were 96.70 ± 35.68 and 83.61 ± 29.73 ml/min/1.73 m^2^, respectively (Table 1).

**Table 1:** **Demographic and clinical characteristics of study participants (n =214)**

Characteristics		
Age (year), means ± SD		45 ± 14.5
Age group, n (%)	18-49 Years	118 (55.2)
	50-59 Years	56 (26.1)
	60-69 Years	28 (13.1)
	≥70 Years	12 (5.6)
Sex, n (%)	Male	123 (57.5)
	Female	91 (42.5)
Type of diabetes, n (%)	Type 1	100 (46.7)
	Type 2	114 (53.3)
Duration of diabetes, n (%)	<5 Years	131 (61.2)
	5-9.9 Years	48 (22.4)
	≥10 Years	35 (16.4)
Family history kidney disease, n (%)		40 (18.7)
Systolic blood pressure (mmHg), means ± SD		121 ± 17
Diastolic blood pressure (mmHg), means ± SD		79 ± 10
Hypertension, n (%)	113 (52.8)	
Body mass index (Kg/m^2^), means ± SD		25.26 ± 4.35
Fasting Serum Glucose (mg/dl), means ± SD		172.85 ± 84.94
Serum Creatinine (mg/dl), means ± SD		1.07 ± 0.33
eGFR _MDRD_ (ml/min/1.73 m^2^), means ± SD)		96.7 ± 35.68
eGFR _C-G_ (ml/min/1.73 m^2^), means ± SD		83.6 ± 29.73

Of the study participants, 18.2% by the MDRD equation and 23.8% by Cockcroft–Gault (C-G) equation had CKD (defined as eGFR *<* 60 ml/min/1.73 m^2^). There was no patient with eGFR <30 ml/min/1.73 m^2^. Only 48.7% and 37.3% of participants with eGFR <60 ml/min/1.73 m^2^ according to the MDRD and C-G equation, respectively had SCr values >1.5 mg/dl (the relevant range for detecting CKD [GFR <60 ml/min/1.73 m^2^]). Table 2 shows the prevalence of CKD according to the K/DOQI classification using the MDRD and C-G equations.

**Table 2:** **Prevalence of CKD according to K/DOQI classification by different equations (n =214)**

Stage	Description	eGFR (ml/min/1.73 m ^2^)	MDRD N (%)	Cockcroft-Gault N (%)
1	Normal or high GFR	≥90	118 (55.1)	84 (39.3)
2	Mild ↓GFR	60-89.9	57 (26.6)	79 (36.9)
3	Moderate ↓GFR	30-59.9	37 (17.3)	49 (22.9)
3A	Mild to moderate ↓GFR	45-59.9	30 (14.0)	32 (15.0)
3B	Moderate to severe ↓GFR	30-44.9	7 (3.3)	17 (7.9)
4	Severe ↓GFR	15-29.9	2 (0.9)	2 (0.9)

An increased risk of CKD was seen with: older age (adjusted OR = 5.30, CI 1.81-15.56); female sex (adjusted OR = 3.34, CI 1.38-8.10); longer duration of diabetes (adjusted OR = 4.08, CI 1.70-9.77); family history of kidney disease (FH-KD) (adjusted OR = 3.16, CI 1.29-7.77); obesity (adjusted OR = 2.75, CI 1.01-7.51) and poor glucose control (high FSG) (adjusted OR = 4.65, CI 1.69-12.76) when renal function was estimated by the MDRD equation. Except for obesity and gender, the same pattern was found when C-G formula was used. The type of diabetes was associated with an increased risk of CKD on multivariate logistic regression when using C-G equation to assess renal function.

From the total study subjects, normal SCr was observed in 193 (90.2%) participants. Of these, 28.5% had mild renal insufficiency (or stage 2 CKD) and 10.4% had moderate renal insufficiency (or stage 3 CKD) despite normal SCr when renal function was estimated by the MDRD equation. When renal function was estimated using C-G equation, 39.9% and 16.6% of those with normal SCr were found to have mild and moderate renal insufficiency or stage 2 and 3 CKD, respectively (Table 3).

**Table 3:** **Kidney function among participants with normal serum creatinine according to the MDRD and Cockcroft-Gault equations (n =193)**

eGFR (ml/min/1.73 m ^2^)	Description	MDRD N (%)	Cockcroft-Gault N (%)
≥90 Stage 1 CKD	Normal renal function	118 (61.1)	84 (43.5)
60 - 89.9 Stage 2 CKD	Mild Renal Insufficiency	55 (28.5)	77 (39.9)
30 - 59.9 Stage 3 CKD	Moderate Renal Insufficiency	20 (10.4)	32 (16.6)

The prevalence of CKD i.e. eGFR <60 ml/min/1.73 m^2^ among participants with normal SCr increased significantly with each age category, from 1.8% for age <49 years to 22.2 and 66.7% for age ≥70 years (all *P* < 0.001) according to the MDRD and C-G, respectively. CKD was also higher in female participants with normal SCr compared to males: 23.5% and 0.0% (*P* < 0.001) by the MDRD, and 27.1% and 8.3% (*P* = 0.001) by C-G.

Compared with patients with eGFR ≥60 ml/min/1.73 m^2^, patients with eGFR <60 ml/min/1.73 m^2^ and normal SCr were older, had high BMI, fasting glucose and serum creatinine when the MDRD equation was used to assess renal function. Similarly, participants with eGFR <60 ml/min/1.73 m^2^ despite normal SCr levels were older, had significantly increased systolic BP, fasting glucose and SCr when renal function was assessed by C-G.

## Discussion

As diabetics are more likely to have CKD than those without diabetics, and thus suffer extra morbidity and mortality; regular assessment of renal function allows early identification of CKD and creates the opportunity for intervention to improve adverse outcomes [2, 10, 17]. Although several studies show a high prevalence of CKD in patients with diabetics, ranging between 15.1–33.1% [16, 19], this study is the first to describe the prevalence of CKD among diabetic adults in Ethiopia using equations to estimate GFR.

In this study, CKD (eGFR *<*60 ml/min/1.73 m^2^) was found in 18.2–23.8% participants depending on the formula used to estimate GFR, with stage 3 being dominant (from 17.3 to 22.9%). Our prevalence estimate of CKD using the MDRD equation was higher than the US NHANES III study [16], which reported 15.1%, and closer to 24.7% reported in Tanzania, sub-Sahara African study using C-G equation [20]. However, our prevalence estimate of CKD using a similar definition of eGFR was lower than that of 27.5% and 31% reported in two UK studies [17, 21], and 33.1% reported in Japanese study [19]. These differences in prevalence might be because of the differences in creatinine assays and calibration or differences in case-mix.

Despite this high prevalence, many patients were undiagnosed because renal function is usually assessed according to the levels of SCr and not to estimate GFR (eGFR). Only 9.8% of the total participants and 48.7% (for the MDRD) and 37.3% (for C-G) of those with eGFR < 60 ml/min/1.73 m^2^ had SCr values >1.5 mg/dl (the relevant range for detecting CKD [GFR <60 ml/min/1.73 m^2^]) [22]. In two different studies, 33 to 45.3% of diabetics with eGFR <60 ml/min/1.73 m^2^ have shown to have raised SCr [17, 21]. Accordingly, SCr failed to detect a significant number of patients with eGFR <60 ml/min/1.73 m^2^.

Analyses presented here also show that, a considerable number of participants ranging from 38.9-56.5% have shown to have mild to moderate RI (stages 2–3 CKD) despite normal SCr levels. A study from Poland also reported a high prevalence of CKD up to 77%, on the basis of eGFR (from 62.8% to 76.9%), among diabetic patients with normal SCr [10]. In our study, CKD i.e. eGFR < 60 ml/min/1.73 m^2^, was found in 10.4% (for the MDRD) and 16.9% (for C-G) of participants with normal SCr. In the above study, 13.0% and 22.4% of diabetics with normal SCr had eGFR < 60 ml/min/1.73 m^2^ according to the MDRD and C-G equations, respectively which is comparable to our results. Thus, depending on normal SCr as evidence of normal renal function will result in missing a substantial proportion of patients with RI.

Furthermore, the rate of undiagnosed CKD defined as eGFR < 60 ml/min/1.73 m^2^ without evidence of abnormal SCr was higher in females and in the elderly. This study shows that the proportion of persons with undiagnosed CKD increased with age; account for the fact that younger people have a higher GFR than older people at the same level of SCr and may translate to late diagnosis of kidney disease. Undiagnosed CKD is also associated with high BMI, fasting glucose and serum creatinine by the MDRD equation, and with high systolic blood pressure, fasting glucose and serum creatinine by C-G equation.

Therefore, as stressed by current guidelines SCr alone should not be used to assess kidney function because it fails to identify many patients whose kidney function is reduced while their SCr is still in the normal range [1, 13]. As SCr is affected by several factors including age, sex, race, and body size, estimation of GFR using prediction equations is recommended to avoid the misclassification of individuals on the basis of SCr alone [14, 15]. Furthermore, it has been shown that the degree of kidney dysfunction in diabetes, as assessed by the eGFR, is an independent predictor of adverse outcomes in these patents [2, 23].

Although several equations that estimate GFR from SCr have been developed, the MDRD and C-G are the commonly used in clinical practice. The MDRD equation that was developed using data from patients with established CKD as measured by ^125^I-iothalamate clearance adjusted for BSA is quick and easy to calculate on all patients using data routinely provided when requesting a SCr measurement. The equation differs from the C-G in several ways including that it does not require knowledge of the patient’s weight and has been generally shown to be more precise and accurate in predicting the GFR in patients with GFR < 60 ml/min/1.73 m^2^ [24, 25]. On the contrary, the C-G equation requires normalisation for BSA, estimates creatinine clearance rather than GFR, and because of the inclusion of weight in the numerator, as a measure of muscle mass, the equation overestimates the GFR in overweight and obese diabetics [26].

Although this study is the first of its type, it has some limitations. First, proteinuria has not been included since a standardized method for measuring albuminuria was not available, thus possibly leading to underestimation of the actual prevalence of CKD. Second, calculated GFR rather than measured GFR was used to diagnose renal insufficiency which is not the gold standard. Third, the MDRD study equation has not been validated among Ethiopian diabetic adults. Lastly, the measurement of serum creatinine was not standardized; this might influence the performance of eGFR equations. Our study also has major strength, the diagnosis of CKD based on eGFR on multiple measures of SCr to establish chronicity.

## Conclusion

In conclusion, this study demonstrated that CKD is present in no less than 18% of diabetic patients attending Butajira hospital of southern Ethiopia, but it is usually undiagnosed. Furthermore, the prevalence of renal insufficiency corresponding to stages 2–3 CKD is high in diabetics despite normal creatinine levels. Therefore, selecting appropriate methods of assessing renal function can identify an important proportion of diabetic patients that are at increased risk for cardiovascular and renal outcomes.
